# Identification of somatic alterations in lipoma using whole exome sequencing

**DOI:** 10.1038/s41598-019-50805-w

**Published:** 2019-10-07

**Authors:** Deepika Kanojia, Pushkar Dakle, Anand Mayakonda, Rajeev Parameswaran, Mark E. Puhaindran, Victor Lee Kwan Min, Vikas Madan, Phillip Koeffler

**Affiliations:** 1 Cancer Science Institute of Singapore, National University of Singapore, Singapore, Singapore; 2 Epigenomics and Cancer Risk Factors, German Cancer Research Center (DKFZ), Heidelberg, Germany; 3 Division of Surgical Oncology, National University Cancer Institute, Singapore, Singapore; 4 Department of Hand and Reconstructive Microsurgery, National University Hospital, Singapore, Singapore; 5 Department of Pathology, National University Hospital, Singapore, Singapore; 6 Division of Hematology/Oncology, Cedars-Sinai Medical Center, University of California, School of Medicine, Los Angeles, California USA; 7 National University Cancer Institute, National University Hospital, Singapore, Singapore

**Keywords:** Cancer genomics, Cancer genomics

## Abstract

Lipomas are benign fatty tumors with a high prevalence rate, mostly found in adults but have a good prognosis. Until now, reason for lipoma occurrence not been identified. We performed whole exome sequencing to define the mutational spectrum in ten lipoma patients along with their matching control samples. We presented genomic insight into the development of lipomas, the most common benign tumor of soft tissue. Our analysis identified 412 somatic variants including missense mutations, splice site variants, frameshift indels, and stop gain/lost. Copy number variation analysis highlighted minor aberrations in patients. Kinase genes and transcriptions factors were among the validated mutated genes critical for cell proliferation and survival. Pathway analysis revealed enrichment of calcium, Wnt and phospholipase D signaling in patients. In conclusion, whole exome sequencing in lipomas identified mutations in genes with a possible role in development and progression of lipomas.

## Introduction

Lipomas, soft tissue fat tumors, are the most common benign mesenchymal tumors^1^. Lipomas are slow growing tumors and often occur under the skin on the neck, shoulders, arms, back, abdomen and thighs. However, occasionally lipomas may be present in deep location or originate within muscle^2^. Lipoma affects only 1% of the population, although it is probably underreported^3^. These benign tumors are commonly found in adults and their prevalence is higher in men than in women. Lipomas are generally small round masses of less than 5 cm and typically are harmless unless they compress an organ. Patients may have either a solitary lipoma or multiple lipomas. Lipomas commonly are diagnosed by clinicians and histologically examined after surgical resection. Interestingly, prognosis for lipomas is very good with rare chance of recurrence after removal of the tumor^4^.

A more aggressive form of fat tumor is liposarcoma, which is also rare in occurrence but frequently develop dangerous metastatic tumors which is symptomatically different from lipomas^4^. Genomic pattern of liposarcomas showed copy number alterations (amplification of Chr 12q) as a major driver of initiation of malignancy along with several putative driver somatic mutations of PI3KCA, TP53, NF1, and EGFR^5–7^. Exact cause of lipomas is still not known, but lipomas have been identified with genetic rearrangements including structural changes at 12q13-15, 13q and 6p21-23 regions^8^. Approximately, 55–75% of solitary lipomas have cytogenetic abnormalities involving *HMGA2* gene rearrangements (truncation or fusion)^9^. Studies revealed enhanced adipogenesis and a normal rate of adipocyte apoptosis in lipoma tissue compared to normal fat tissue^10^. Lipoma appears to be close to well-differentiated liposarcomas in terms of their HMGA2 structural alterations and high adipogenicity^11^. However, no convincing literature existed to show liposarcomas arises from lipomas.

Genomic characterization of tumors improved our understanding of their molecular genesis for better management of the disease. Therefore, we screened superficial subcutaneous lipomas using whole exome sequencing to systematically profile their mutational signatures, copy number variations and identify affected pathways for better understanding of their molecular pathogenesis.

## Results and Discussion

We performed a genomic analysis to profile the somatic mutational landscape of lipomas by whole exome sequencing. To best of our knowledge, this is first study to perform exome sequencing on lipomas.

### Clinical characteristics of lipoma patients

Primary subcutaneous lipomas [ordinary lipomas] were obtained from patients with their consent undergoing surgical resection at the National University Hospital. Detailed clinical features associated with these benign tumors presented in Supplementary Table 1. We collected lipomas from ten patients (all males) with a median age of 52 years. We performed whole exome sequencing on ten lipomas and normal controls from the same patient (either blood sample or adjacent normal tissue). Detail of normal controls used for each patient is given in Supplementary Table 1. Lipomas generally include various histologic subtypes but the samples used in the study are the common superficial primary subcutaneous solitary lipomas found on either back or arm.

### Sequencing data

Whole exome sequencing of lipoma samples achieved an average of 135 million reads per sample (range 111–188 million). The control samples from the same patients found to have an average of 128 million reads per sample (range 112–164 million). Sequencing obtained a mean depth of 93 and 85 across the sequencing regions in lipomas and matching controls, respectively.

Allele frequency (AF) implicate how early a mutation occurred during progression of the tumor and our data suggest that the mutations occurred both early (variants with high AF) and later (variants with low AF) during development of their lipoma. In total, 5 frameshift deletions and 5 frameshift insertions occurred with 5–20% AF.

### Mutational landscape in lipomas

**Figure 1:**
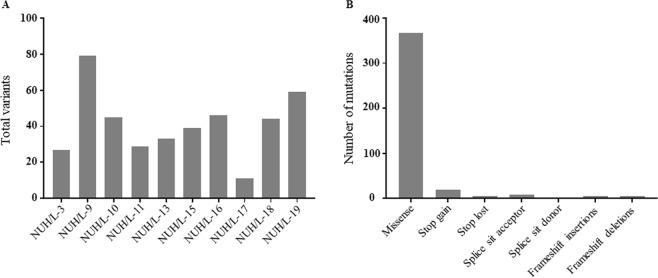
Variants identified in ten lipomas using whole exome sequencing. (**A**) Total number of variants found in each lipoma sample. **(B)** Number of each type of variant identified in ten lipoma samples.

Using our data pipeline analysis, we identified 412 somatic variants with a median of 42 per patient sample (range 11–79) [Fig. 1A and Supplementary Tables 2–11]. The type of mutations include 367 missense mutations, 19 stop gained, 5 stop lost, 9 splice acceptor, 2 splice site donor, 5 frameshift insertions and 5 frameshift deletions [Fig. 1B]. Details of types of mutation for each sample listed in Supplementary Table 12. Similar to other benign tumors and sarcomas, overall mutation burden in lipomas observed to be relatively low.

We validated the variants identified by data analysis of whole exome sequencing using Sanger sequencing as secondary technology to avoid the possibility of reporting out a false-positive result. However, Sanger sequencing has a limit of detecting somatic variants with AF about 10%, and higher. Variants with more than 10% of AF subjected to Sanger sequencing. We validated 42 variants out of 129 in total confirming these as true positive mutations in the patients. These 42 variants included 37 missense mutations, 1 frameshift deletion, 2 in-frame deletions, 1 splice donor and 1 stop gained mutation. Detailed list of variants, their mutation type and known function of genes provided in Supplementary Table 13. Validated mutations included kinase genes; ACVRIC (AF 28%), PTPRT (AF 14%), ERBB2 (AF 14%) and MAPK7 (AF 20%) and transcription factors ATMIN (AF 7%), ZNF317 (AF 26%) and FOSL1 (AF 19%); each are crucial for cell survival and proliferation. No single mutated gene was common in all ten tumors. Several of the genes are known to play functional role in oncogenesis such as APC (AF 26%), FOSL1 (AF 19%), ERBB2 (AF 14%), NFATC3 (AF 10%), PDE1A (AF 11%), ATMIN (AF 7%), and MAPK7 (AF 20%). In addition, experimental evidence have also indicated modulation of FOSL1^12^, ERBB2^13^, and PDE1A^14^ regulates adipogenesis.

We also explored the differences or similarities of lipomas with cancerous liposarcomas and found few similarities with malignant liposarcomas. We compared the mutations identified in lipomas with mutation data of several liposarcoma studies^5,15,16^ and found three genes APC, RYR2 & MAPK7 mutated in both lipomas and liposarcomas. We also screened gene variants mutated in lipomas in sarcomas TCGA database and observed majority of these genes showed copy number alterations with very few mutations. However, variants identified in lipomas are different from sarcomas and not driver variants causing the disease. Therefore, we could not inferred any relation between lipomas and liposarcomas as it is difficult to interpret based on few cases of lipomas with no recurrent alterations in lipomas.

### Copy number variation analysis in lipoma patients

PureCN Rpackage was used to calculate the tumor purity and ploidy to call copy number in lipoma samples^17^. The samples used for whole exome sequencing were overall not highly pure, and two samples were flagged by PureCN. The tumor purity and ploidy calculated for each patient samples given in Supplementary Table 14. Due to very low tumor purity of sample NUH/L-3, we excluded the sample from this analysis. Low tumor purity affected the allelic frequency of the variants identified in tumors in our study and decreased sensitivity of detection. The tumor samples used in our study found to be contaminated with normal probably due to small size of these tumors, little control over purity at clinic level and few obtained from biopsy samples instead of surgical resections.

**Figure 2:**
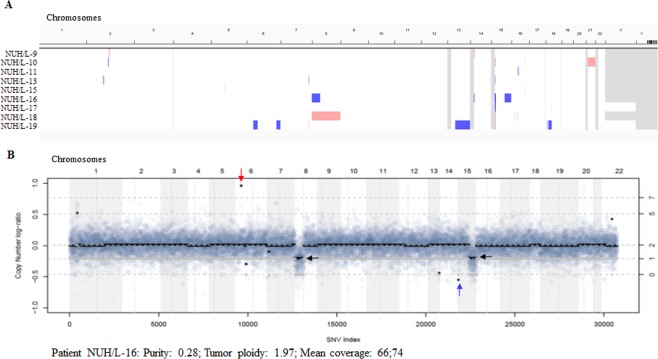
Copy number alterations in lipoma samples. (**A)** Genome-wide copy number variations in lipoma samples showing minor alterations. Chromosomes are represented from left to right in the order. Light blue indicates LOH (or shallow deletion), light red indicates three copies gain, and dark red indicates copy number equivalent to more than three copies. **(B)** Copy number log-ratios of sample NUH/L-16 analysed by PureCN. A dot represents a germline SNP and background colors visualize chromosomes and vertical dotted lines centromere positions. Grey line represents the expected allelic fractions in the segment, which are calculated using the estimated purity and segment copy numbers. Red arrow marks amplification, blue arrow marks deletion and black arrow marks LOH.

Copy number analysis indicated lipomas are mostly genomically silent as only few minor copy number aberrations were observed in these patients [Fig. 2A]. These results are in concordant with low mutation burden in lipoma samples. Analysis identified few amplifications, deletions and loss of heterozygosity (LOH) in lipoma patients, which are tabulated in Supplementary Tables 15 and 16. As a representative, copy number of sample NUH/L-16 presented as log-ratio demonstrating amplification (red arrow), deletion (blue arrow) and LOH (black arrow) [Fig. 2B]. Plots of copy number log-ratio of all other samples shown in Supplementary Fig. 1. Three recurrent copy number changes found in lipoma samples; deletion of NFSAP1, LINC02203 and OR4M2 genes in two patients. Few LOH regions were observed in two or more than two lipoma patients such as chr15:20169937-21236751, chr6:31948633-32014371, and chr8:7272427-7754188. List of LOH regions identified in patient samples provided in Supplementary Table 16.

### Mutational patterns in lipoma patient samples

**Figure 3:**
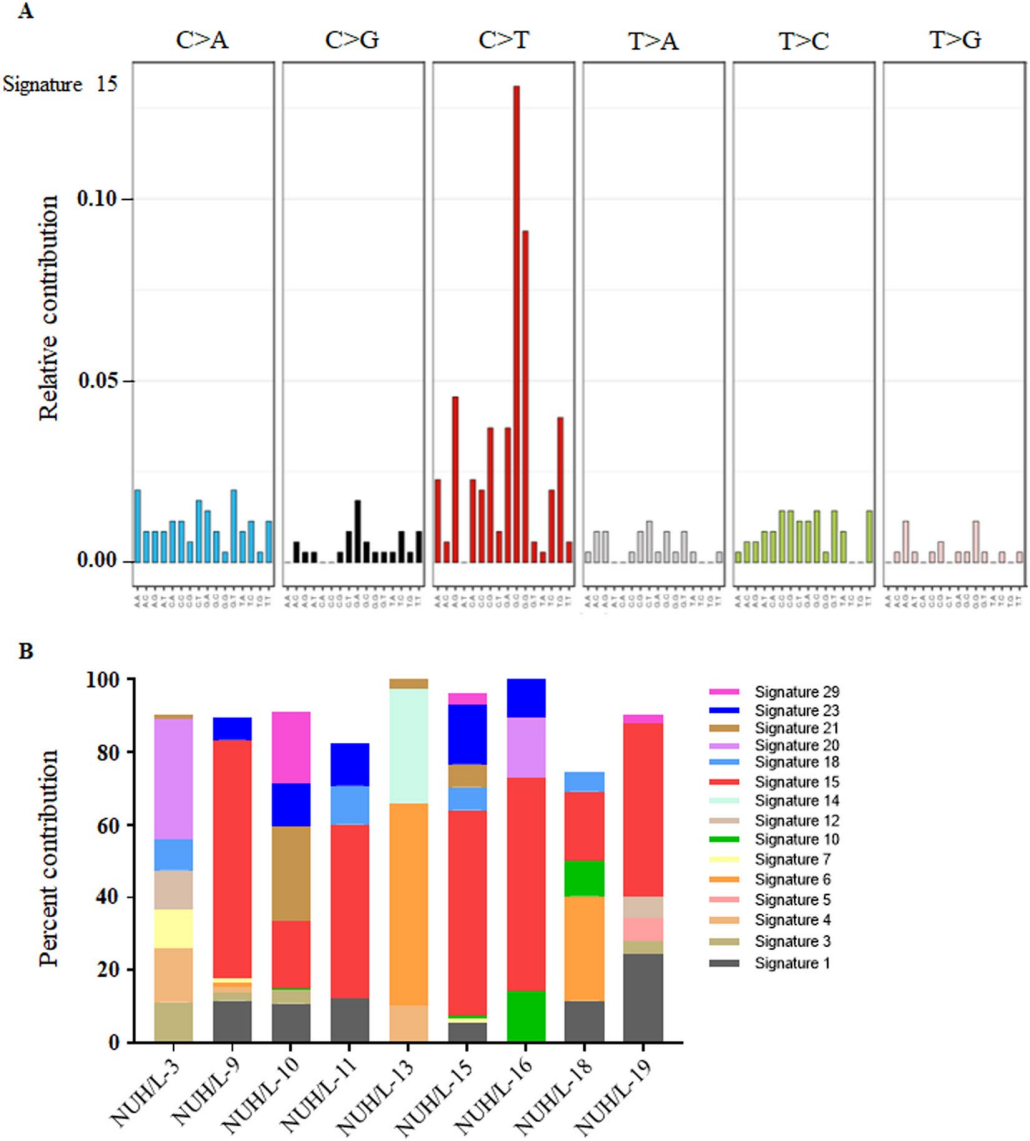
Signature of mutational process in lipomas. (**A)** Mutational signatures presented according to 96-substitution classification highlighting signature 15 as major process associated with lipomas. **(B)** Histogram shows percentage of signatures present in each lipoma sample.

We performed mutational spectrum analysis of lipomas to categorize their mutational signature and to identify functional mutagenic processes in lipomas. Patient wise signature analysis is shown in Supplementary Fig. 2. No mutational signature was detected for patient NUH/L-17 due to lower mutational burden and low allele frequency of variants. Analysis revealed mutational signature 15 as the major signature affecting lipoma patients [Fig. 3A,B]. Signature 15 is one of the mutational signature associated with defective DNA mismatch repair that contributes to large numbers of substitutions and small indels at nucleotide repeats. In addition, this signature found in several samples of lung and stomach cancer. Surprisingly, this is the most common signature in a benign tumor. We had an interesting observation that mutational signature 1 affects malignant liposarcomas in previous study^5^ whereas none of the lipoma patients showed this signature. Therefore, these results suggests different biological mechanisms involved in mutagenesis of lipoma.

### Altered pathways in lipomas

Pathway analysis was conducted to identify pathways and biological processes affected by mutated genes in lipomas. This analysis included gene variants validated and confirmed with Sanger sequencing. KEGG analysis indicated that out of 42 validated genes, eleven were involved in signalling of calcium (*P* = 0.0005), Wnt (*P* = 0.003), phospholipase D (*P* = 0.03) as well as stem cells’ pluripotency (*P* = 0.003) and axon guidance (*P* = 0.03) pathway [Supplementary Fig. 3A]. In addition, biological processes were identified which were associated with genes mutated in lipoma samples including calcium ion transport and microtubule regulation as well as signalling through the insulin receptor and tyrosine kinase [Supplementary Fig. 3B]. Interestingly, calcium signalling pathway and calcium ion transport biological process emerged as a major pathway/process affected by mutated genes in lipoma which were largely unaffected in liposarcoma patients’ exome study^5^. Several studies have indicated a regulatory role of calcium ions in adipogenesis contributing to increased fat tissue by promoting and accelerating pre-adipocyte differentiation^18,19^, which is a feature of lipomas. In addition, Wnt signaling^20^ and phospholipase D signalling^21^ pathways shown to regulate adipogenesis.

In conclusion, we identified and validated somatic mutations in ten lipoma patient samples using whole exome sequencing analysis. However, recurrent gene variants and copy number variations not noted in lipoma patients; thus suggesting these alterations could be passenger events. Additional samples need to be sequenced and the functional consequences of the identified mutations need to be explored to support the present findings. Present analysis also delineated lipoma-specific mutational signatures and identified wide-ranging genomic landscape of altered genes and pathways in lipomas that could be useful to investigate further the functional cause of lipoma and its progression.

## Methods

### Tissue collection and sample preparation

Lipomas, normal adjacent control tissues and patient’s blood samples were obtained from National University Hospital, Tissue repository, Singapore in accordance with ethical guidelines of National University of Singapore, Institutional Review Board (NUS-IRB). According to their requirement, samples were collected after obtaining informed written consent from patients. Tissue samples or blood received after surgery was snap frozen and stored in liquid nitrogen until processing. Genomic DNAs were isolated from tumors, control tissues and blood samples using QIAamp DNA Mini Kit (Qiagen) according to the manufacturer’s instructions. Genomic DNAs were quantified by Qubit dsDNA BR assay kit (Life technologies) and their quality verified by agarose gel electrophoresis. All the experimental protocols were approved by the National University of Singapore, Office of safety, health and environment (NUS, OSHE).

### Whole exome sequencing

Exome libraries were prepared using the Agilent SureSelect All Human Exon v5 capture kit^5^. Briefly, the qualified genomic DNA samples were randomly fragmented into base pair peak of 150 to 200 bp, and adapters ligated to both ends of the resulting fragments. Adapter-ligated templates were purified by the Agencourt AMPure SPRI beads, and fragments with insert size of about 200 bp were excised. Extracted DNA was amplified by ligation-mediated polymerase chain reaction (LM-PCR), purified, and hybridized to the SureSelect Biotinylated RNA Library (BAITS) for enrichment. Hybridized fragments were bound to the streptavidin beads whereas non-hybridized fragments were washed away after 24 h. Captured LM-PCR products were subjected to Agilent 2100 Bioanalyzer to estimate the magnitude of enrichment. Each captured library was independently loaded on Hiseq 4000 platform for high-throughput sequencing to ensure that each sample meets the desired average fold-coverage. Raw image files were processed by Illumina base calling Software 1.7 with default parameters, and the sequences were generated as 90 bp paired-end reads. All the experimental protocols were approved by the NUS, OSHE.

### Analysis of whole exome sequencing

Paired end sequencing reads were aligned to the human reference genome (hg19) using bwa mem. Samblaster was subsequently used to mark PCR duplicates^22^. Systematic errors in base quality scores were then corrected using GATK4 Base Recalibrator^23^. Somatic single nucleotide variants and short indels were detected against a matched normal using both GATK4 Mutect2 and Strelka2^24^. Variants calls from Mutect2 further filtered with GATK4 FilterMutectCalls. vcf2maf was used to convert the .vcf files to MAF format and also annotated using Ensembl VEP (Version 92_GRCh37)^25^. Depth of coverage for all samples calculated using GATK3 DepthOfCoverage.

We excluded any variants which were not tagged PASS by FilterMutectCalls or Strelka2. Variants were further filtered to remove those with global minor allele frequency (GMAF) lower than 1% or variants tagged as common variants by vcf2maf based on allele count in Exac populations, while still retaining those classified as “pathogenic” in ClinVar. Variants with either LOW/MODIFIER impact as per VEP or with variant AF less than 0.01 were also removed.

### Mutation signature analysis

MuSiCa, online tool was used for somatic characterization of mutation signatures present in the patient’s variants list^26^. This tool based on MutationalPatterns and built using the Shiny framework in R language. MutationalPatterns extracts de novo signatures using the original NMF algorithm and quantifies COSMIC-reported signatures. A series of mutational profiles extracted using the information of somatic single nucleotide variants and 96 possibilities assessed to detect processes accountable for the same substitutions but in different contexts.

### Pathway analysis

Refseq-annotated mutations (genes) were used for pathway analysis using PathScan algorithm integrated into MuSic pipeline^27,28^. P values calculated in KEGG pathway analysis, where, first it identifies the subset of genes, and proteins, that contribute in a particular KEGG pathway for the input. Then, it evaluates the fraction of those biological features that overlaps with the set of features of input genes. In the final step, KEGG computes the significance of the overlap using the Fisher exact test, which represents as p-value interpreted as a measurement of the confidence that this overlap is due to chance.

### Copy number variation analysis

PureCN^17^ was used to estimate tumor purity and call copy number alterations. Coverage values for the targeted regions were calculated and normalized for GC-content and replication bias. Replication timing data for K562 cell line (wgEncodeUwRepliSeqK562WaveSignalRep1.bigWig) was obtained from the UCSC genome browser. A variant caller format (VCF) file containing germline variants present in 3 or more normal samples was used for obtaining read mapping bias estimates. Heterozygous short-nucleotide polymorphisms (SNPs) in targeted regions including 50 bp flanks were used to calculate the allele-specific copy numbers. Genes were called amplified if the segment copy number was ≥6 for focal amplifications or ≥7 for non-focal amplifications. Genes with a segment copy number of ≤0.5 were called as deletions.

### Sanger sequencing validations

Gene variants with AF greater than 10% were validated by Sanger sequencing. Polymerase chain reaction was performed using 25 ng template DNA of lipoma tissue and normal control sample from the same individual. PCR products run on 1% agarose gel and purified using Wizard® SV Gel and PCR Clean-Up System (Promega). Purified PCR products were sequenced and analysed with the Sequence Scanner Software 2 Version 2 (Applied Biosystems).

### Ethics approval and consent to participate

Patient’s tissues and blood samples collected in accordance with ethical guidelines of National University of Singapore, Institutional Review Board (NUS-IRB) and after obtaining informed written consent from patients. All the experimental protocols were approved by the National University of Singapore, Office of safety, health and environment (NUS, OSHE).

## Supplementary information

Supplementary Figures

Supplementary Tables
