# Supplementary material for: Identification of somatic alterations in lipoma using whole exome sequencing
Source: Sci Rep. 2019 Oct 7;9:14370. doi: 10.1038/s41598-019-50805-w (PMC6779901; doi:10.1038/s41598-019-50805-w)
Supplement: Supplementary file 1 — Supplementary Figures [file 41598_2019_50805_MOESM1_ESM.pdf]

Supplementary Information

Identification of somatic alterations in lipoma using whole exome sequencing

Deepika Kanojia[1*], Pushkar Dakle[1], Anand Mayakonda[1,2], Rajeev Parameswaran[3], Mark E Puhaindran[4], Victor Lee Kwan Min[5], Vikas Madan[1] and Phillip Koeffler[1,6,7]

[1]Cancer Science Institute of Singapore, National University of Singapore, Singapore; [2]Epigenomics and Cancer Risk Factors, German Cancer Research Center (DKFZ), Heidelberg, Germany; [3]Division of Surgical Oncology, National University Cancer Institute, Singapore; [4]Department of Hand and Reconstructive Microsurgery, National University Hospital; [5]Department of Pathology, National University Hospital; [6]Division of Hematology/Oncology, Cedars-Sinai Medical Center, University of California, School of Medicine, Los Angeles, California, USA; [7]National University Cancer Institute, National University Hospital, Singapore.

***Correspondence:** csidk@nus.edu.sg

Supplementary Figure 1

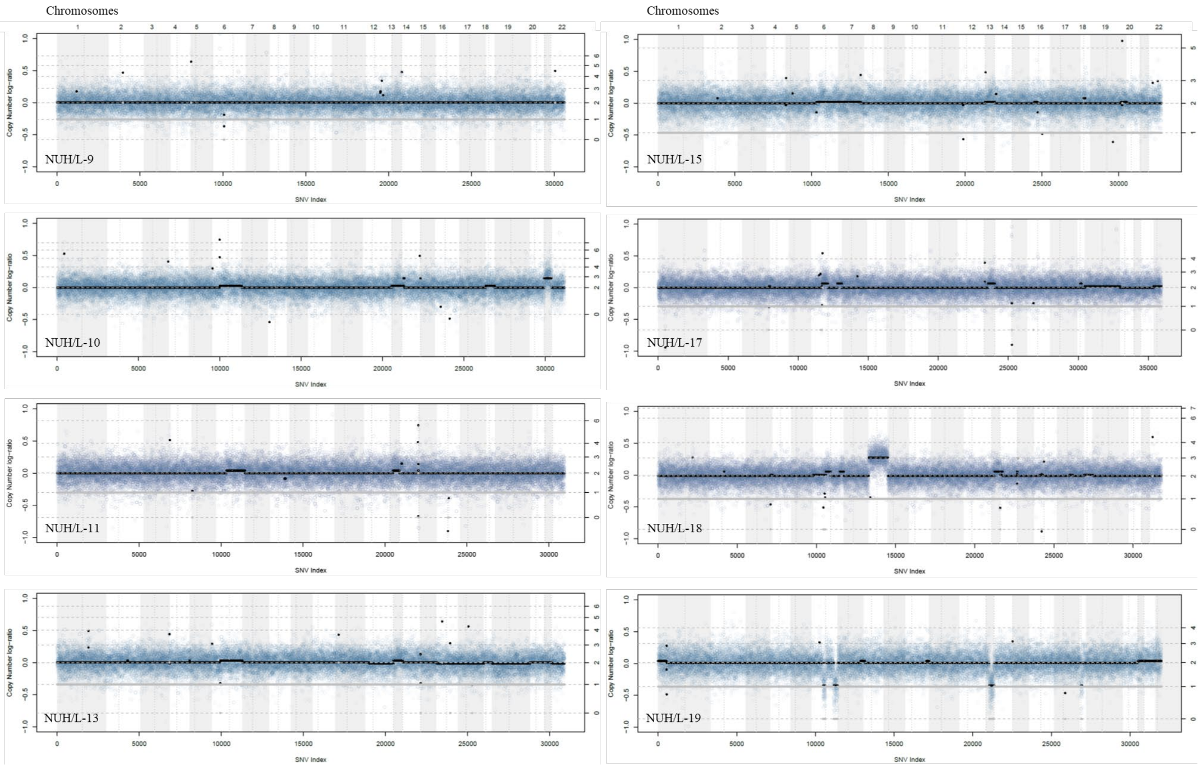

Supplementary Figure. 1: Copy number log-ratios of sample NUH/L-16 analysed by PureCN Rpackage. A dot represents a germline SNP and background colors visualize chromosomes and vertical dotted lines centromere positions. Grey line represents the expected allelic fractions in the segment, which are calculated using the estimated purity and segment copy numbers.

Supplementary Figure 2

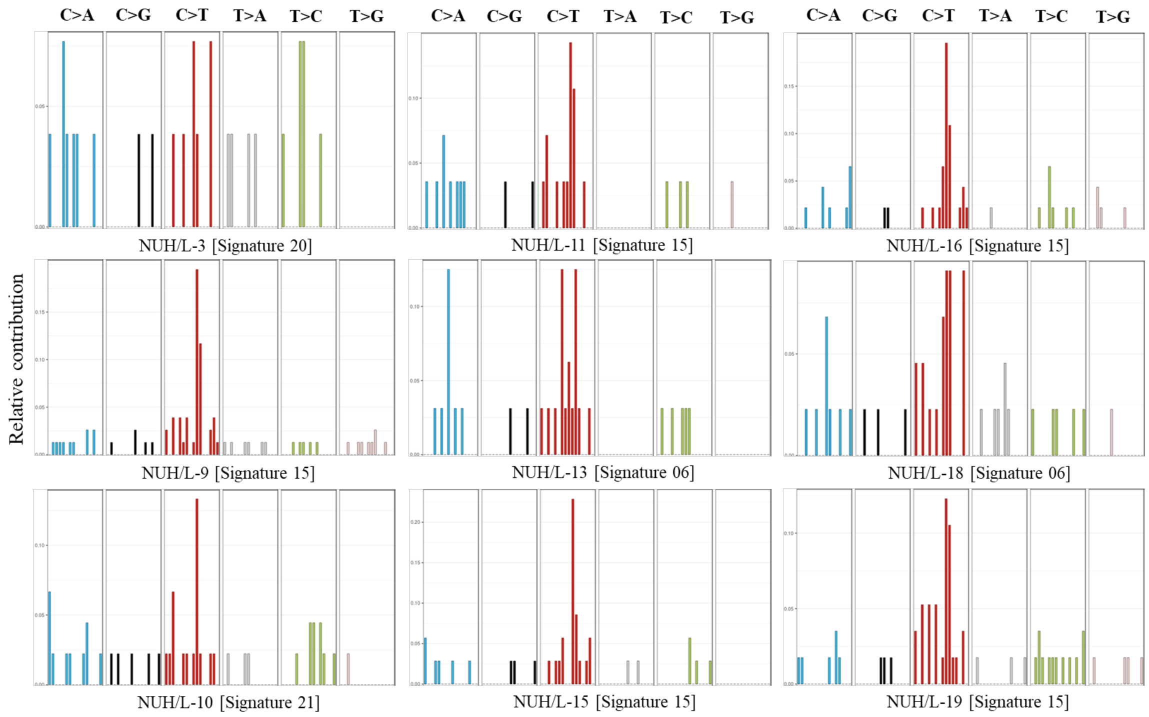

Supplementary Figure. 2: Mutation signature associated with each lipoma patient.

Supplementary Figure 3

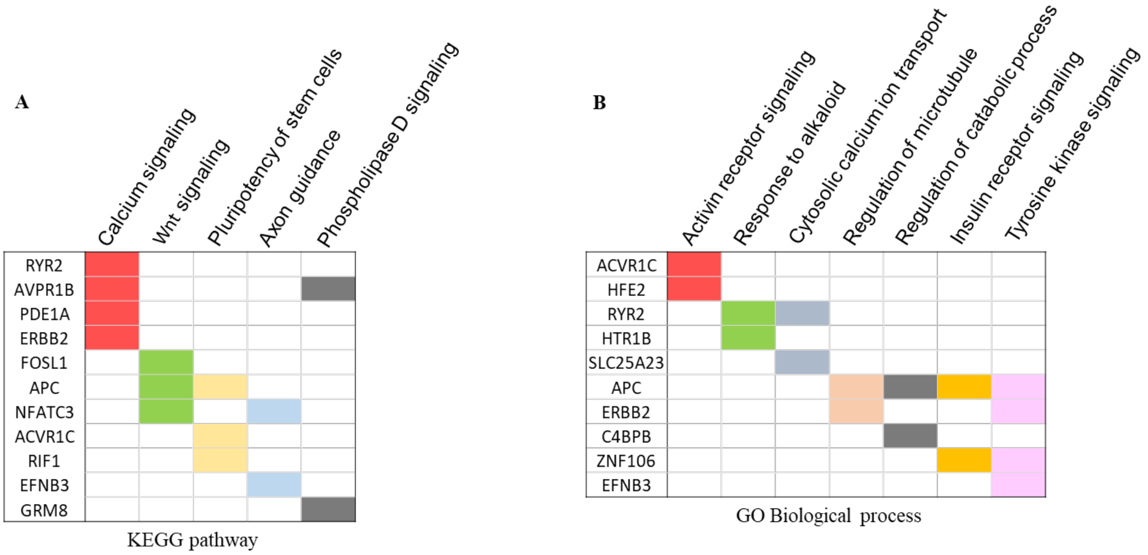

Supplementary Figure. 3: Enriched pathways (A) and biological processes (B) identified and associated with validated mutated gene products in lipoma tumors.
